# Enhancing the Thermo-Stability and Anti-Biofilm Activity of Alginate Lyase by Immobilization on Low Molecular Weight Chitosan Nanoparticles

**DOI:** 10.3390/ijms20184565

**Published:** 2019-09-14

**Authors:** Shangyong Li, Yanan Wang, Xiao Li, Beom Suk Lee, Samil Jung, Myeong-Sok Lee

**Affiliations:** 1 Department of Pharmacology, College of Basic Medicine, Qingdao University, Qingdao 266071, China; lisy@qdu.edu.cn (S.L.); sunshine4581@163.com (Y.W.); lilix0823@163.com (X.L.); 2 Molecular Cancer Biology Laboratory, Cellular Heterogeneity Research Center, Department of Biosystem, Sookmyung Women’s University, Hyochangwon gil-52, Yongsan-Gu, Seoul 140-742, Korea; min9996@nate.com (B.S.L.); samiljung@sookmyung.ac.kr (S.J.)

**Keywords:** immobilization, chitosan nanoparticles, alginate lyase, anti-biofilm activity, antibiotics susceptibility

## Abstract

Bacterial biofilm causes severe antibiotic resistance. An extracellular polymeric substance (EPS) is the main component in the bacterial biofilm. Alginate is a key EPS component in the biofilm of *Pseudomonas aeruginosa* and responsible for surface adhesion and stabilization of biofilm. Alginate lyase has emerged as an efficient therapeutic strategy targeting to degrade the alginate in the biofilm of *P. aeruginosa*. However, the application of this enzyme is limited by its poor stability. In this study, chitosan nanoparticles (CS-NPs) were synthesized using low molecular weight chitosan and alginate lyase Aly08 was immobilized on low molecular weight chitosan nanoparticles (AL-LMW-CS-NPs). As a result, the immobilization significantly enhanced the thermal stability and reusability of Aly08. In addition, compared with free Aly08, the immobilized AL-LMW-CS-NPs exhibited higher efficiency in inhibiting biofilm formation and interrupting the established mature biofilm of *P. aeruginosa*, which could reduce its biomass and thickness confirmed by confocal microscopy. Moreover, the biofilm disruption greatly increased the antibiotic sensitivity of *P. aeruginosa*. This research will contribute to the further development of alginate lyase as an anti-biofilm agent.

## 1. Introduction

Bacterial biofilms are generally defined as structured, specialized and adherent microbial communities. The presence of biofilm reduces the sensitivity of bacteria to conventional antibiotics around 1000-fold compared with planktonic ones [1,2,3]. The complex extracellular polymeric substance (EPS) produced by microbial cells is considered to play important roles in adhesion and aggregation of bacterial biofilm, mediating cell–cell and cell–surface connectivity, as well as protecting bacteria escape from the immune system of the host [4,5]. Recent estimates suggest that biofilms are accounting for over 80% of microbial infections, however, the traditional antibacterial agents were more effective towards free bacteria than biofilm embedded microbes due to its impaired penetration in biofilm. In addition, the problem of bacterial resistance to antibiotics has attracted increasing attention [6,7]. Thus, it is crucial to exploit new strategies which simultaneously destabilize the biofilm architecture and ensure safety.

Thus far, many strategies have been introduced in order to kill pathogenic bacteria in biofilms, such as the application of weak organic acids (WOA), bacteriophage, antimicrobial peptides (AMPs), iron chelation, mutating biofilm development by interfering with the signaling pathways involved, and photo irradiation [8,9,10,11]. The application of bacteriophage in the treatment of *Pseudomonas aeruginosa* infections as an alternative to antibiotics has attracted attention and there are 137 different phages targeting the *Pseudomonas* genus that have been characterized, but it faced the problems of bacterial resistance and poor stability [12,13]. Numerous AMPs, including cecropin P1, magainin II, LL37, and so on, target bacterial cytoplasmic membrane, causing cell death [14,15]. Contrarily, the disadvantages of AMPs are hemolytic, proteolytic sensitivity, and high cost [16]. In addition, the problems in clinical application of WOA includes how to choose the acid effectively against target species and the unknown antibacterial mechanism. There are also significant potential problems with cytotoxicity from the treatments of photo irradiation and iron chelation. Among the different approaches, the using of biodegradable enzyme to remove biofilm by destroying the EPS combined with antibiotics has been proven to improve the bacterial sensitivity to antimicrobials as compared to antibiotics alone [17,18,19].

*P. aeruginosa*, an opportunistic human pathogen, is a major causative agent of mortality and morbidity in immunocompromised individuals and those with cystic fibrosis (CF). *P. aeruginosa* invades the lung epithelia and shows the genetic transformation from non-alginate producing (nonmucoid) to alginate producing strain (mucoid), which supports higher bacterial adherence in lung mucosa, stabilization of biofilm and immune escape [20,21]. Alginate is an acidic heteropolysaccharide, consisting of β-d-mannuronate (M) and α-l-guluronate (G), which is a key EPS component in the biofilm of *P. aeruginosa* [22]. Therefore, targeting to disrupt the alginate in the biofilm by using alginate lyase has emerged as an efficient therapeutic strategy. Although, various of alginate lyases have been cloned and characterized, the research on its anti-biofilm activity are rather rare, and the primary limitation of application of this enzyme is its instability. To effectively improve the stability and reusability of the alginate lyase, the implementation of immobilization of enzymes strategy has attracted more attention. Chitosan is widely available, has a low-cost and better biodegradability and biocompatibility, better ability for loading large amounts of enzyme and nontoxicity, thereby, it is well established as a suitable matrix for enzyme immobilization compared to native biomolecules [23,24]. Moreover, the natural biopolymer, chitosan, has been shown to have broad-spectrum antimicrobial activity without increasing resistance [23]. Recently, alginate lyase immobilized chitosan nanoparticles (CS-NPs) of ciprofloxacin has showed improved antimicrobial activity against the biofilm associated mucoid *P. aeruginosa* infection in CF [21].

In our previous study, an alginate lyase, Aly08, was cloned from marine bacterium *Vibrio* sp. SY01, which can effectively perform alginate degradation effects [25]. In order to improve its biological properties, alginate lyase Aly08, immobilized on low molecule weight (LMW) CS-NPs, was detected. Compared with the free enzyme, the biological properties of the immobilized enzyme were significantly enhanced, such as thermal stability and reusability. This study also showed that the immobilized enzyme was more effective than the free enzyme in inhibiting biofilm formation and interrupting the mature biofilm towards *P. aeruginosa*. Moreover, the biofilm disruption greatly increased the antibiotic sensitivity of *P. aeruginosa*. Thus, CS-NPs will be helpful for the further development of alginate lyase as an anti-biofilm agent.

## 2. Results and Discussion

### 2.1. Synthesis and Determination of Chitosan Nanoparticles

As one of the most abundant natural polymers on the earth, chitosan and its synthetic nanoparticles have been widely used in biomedicine. The physicochemical properties of CS-NPs are very different depending on the origin of chitosan and the processing method. The high molecular weight chitosan (HMW-CS) was hardly dissolved in the neutral condition. The preparation process of high molecular weight chitosan nanoparticles (HMW-CS-NPs) required a large amount of HCl treatment, which significantly restricted its practical application. Meanwhile, the molecular weight of chitosan significantly influenced the size and size dispersion of CS-NPs. Compared with HMW-CS, low molecular weight chitosan (LMW-CS) showed many advantages in CS-NPs production, such as better water solubility, smaller size, and higher drug loading. Thus far, LMW-CS was produced by several methods: physical degradation method (microwave and ultrasonication), chemical degradation method (acidic and alkaline treatment), and enzymatic method [26,27]. In contrast to the physical and chemical methods, enzymatic production method is advantageous in moderate reaction conditions, a lower risk of environmental pollution and easily-controlled product [28]. Lysozymes and chitosanases are two principal widely used enzymes in terms of enzymatic method of LMW-CS producing [29,30]. Lysozymes hydrolyze chitosan non-specifically and inefficiently. Compared with lysozymes, chitosanases hydrolyze chitosan with higher efficiency, however, the cutting segments of which are generally too small to control the reaction conditions readily [31]. Herein, a rapid and efficient LMW-CS producing protocol was developed using the chitosanase CsnM purified in our lab [32]. In our previous study, CsnM showed the cold-adapted property. The reaction was controlled easily. When the reaction temperature was above 40 °C, the enzyme lost its activity immediately. According to the viscosity analysis, the average molecular weight of the LMW-CS used in this study is 8.76 kDa, which was suitable for synthesis of low molecular weight chitosan nanoparticles (LMW-CS-NPs).

**Figure 1:**
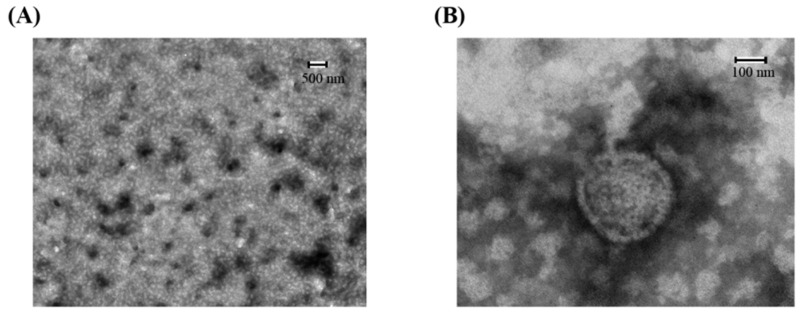
Transmission electron microscopy (TEM) micrograph of nanoparticles at 500 nm (**A**) and 100 nm (**B**). The samples were immobilized on copper grids and dried at room temperature. Then they were stained with phosphate tungsten acid and examined by TEM.

Transmission electron microscopy (TEM) was performed to examine the morphology and size of the synthetic LMW-CS-NPs (Figure 1). TEM images exhibited that the CS-NPs have almost spherical shape and smooth surface, and the size of the nanoparticles ranges from 100 to 300 nm. As a control, the diameter of the CS-NPs prepared by HMW-CS at the same reaction condition is generally greater than 500 nm (Figure S1).

In this study, the alginate lyase Aly08 was used for synthesizing the alginate lyase immobilized low molecular weight chitosan nanoparticles (AL-LMW-CS-NPs). Aly08 was a typical endo-type alginate lyase, which had been fully characterized in our lab [25]. In addition to the above water-soluble and low-size advantages, LMW-CS-NPs exhibited a growing number of chains at the same quality leading to more free amino groups on the surface to immobilize protein. According to the ninhydrin analysis, the number of free amino acid on the surface of HMW-CS-NPs is only 27.6% of the LMW-CS-NPs.

**Figure 2:**
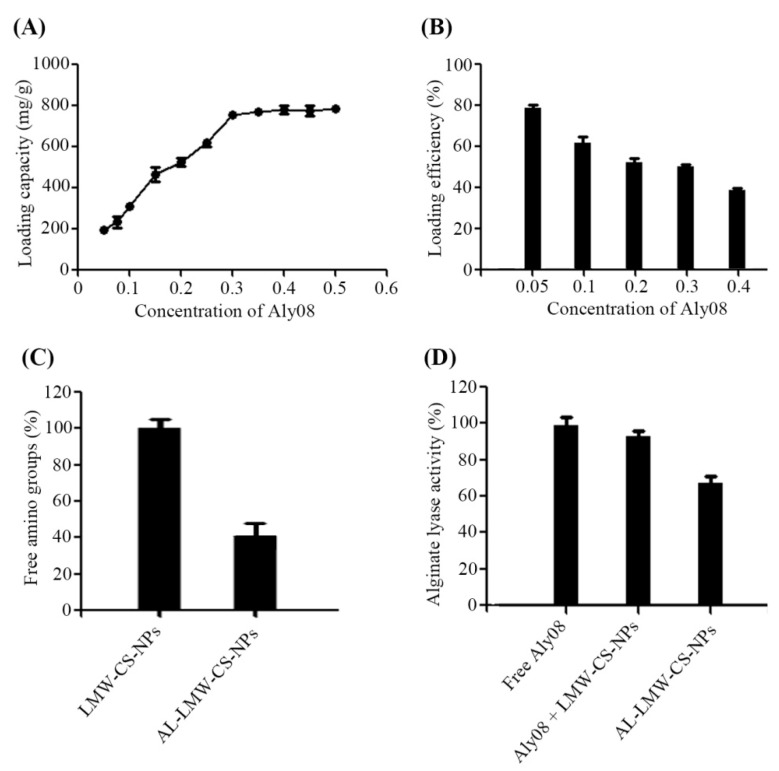
Analysis of alginate lyase immobilized low molecular weight chitosan nanoparticles (AL-LMW-CS-NPs). (**A**) Aly08 loading capacity of AL-LMW-CS-NPs; (**B**) Aly08 loading efficiency of AL-LMW-CS-NPs; (**C**) The % free amino groups of before (LMW-CS-NPs) and after (AL-LMW-CS-NPs) Aly08 loading; (**D**) The % alginate lyase activity of different groups.

The loading capacity (LC) of Aly08 was directly affected by its concentration (Figure 2A). The LC of Aly08 was apparent increase from 192.3 to 787.3 mg/g, when the Aly08 concentration was in the range of 0.05–0.30 mg/mL. After the concentration exceeds 0.3 mg/mL, the LC of Aly08 only increased slowly. As shown in Figure 2B, the loading efficiency (% LE) reduced from 79.3% to 37.5% as the concentration of Aly08 increased from 0.05 to 0.40 mg/mL. These findings suggested that the AL-LMW-CS-NPs used in this study were almost saturated at 0.30 mg/mL. As shown in Figure 2C, 40.2% free amino group was determined on the surface of AL-LMW-CS-NPs after Aly08 loading (0.3 mg/mL), indicating that approximately 59.8% amino groups of LMW-CS-NPs conjugated with the Aly08. To determine the enzymatic activity of the synthetic AL-LMW-CS-NPs, free Aly08 with the same protein concentration was used as a control. As shown in Figure 2D, the immobilized AL-LMW-CS-NPs showed an effective activity (68.7%). The free Aly08 combined with water-soluble LMW-CS-NPs only slightly influence the activity of Aly08.

### 2.2. Biochemical Characterizartion of AL-LMW-CS-NPs

**Figure 3:**
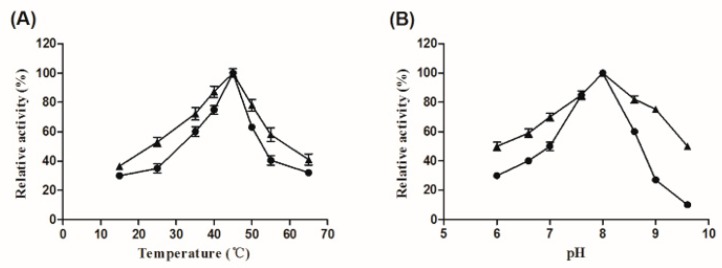
Effect of temperature (**A**) and pH (**B**) on the activity of free (circles) and immobilized (triangles) Aly08. (**A**) Optimal temperature for the activity of free and immobilized Aly08 was determined in 50 mM sodium phosphate buffer (pH 8.0) at diverse temperatures (15–65 °C). (**B**) Optimal pH for the activity of free and immobilized Aly08 was determined at 45 °C. The enzymatic activity of a fresh sample of free or immobilized Aly08 measured at pH 8.0 and 45 °C was defined as 100%.

The characterizations of the free and immobilized Aly08 were further analyzed. As shown in Figure 3A, the enzymatic activity of the free and immobilized Aly08 were increased from 15 °C and then reached a peak at 45 °C, afterwards dropped with further increase of temperature. As a result, the free and immobilized Aly08 both showed the optimum temperature at 45 °C. As shown in Figure 3B, the optimal reaction pH of both the free and immobilized Aly08 were 8.0. These results indicated that the immobilization of Aly08 on LMW-CS-NPs does not influence its optimum temperature and pH.

**Figure 4:**
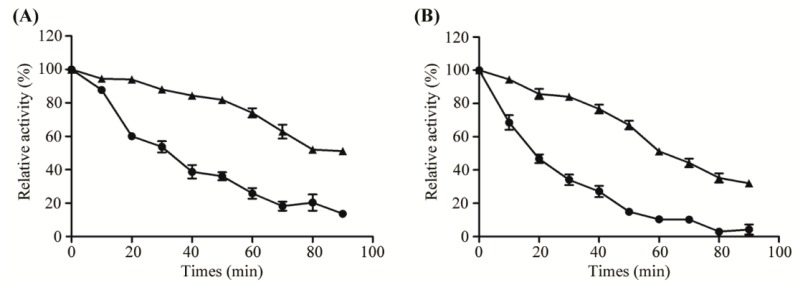
Thermo-stability of free (circles) and immobilized (triangles) Aly08 at 37 °C (**A**) and 45 °C (**B**). Residual activities were measured at 45 °C and pH 8.0. The activity of enzyme without incubation was defined as 100%.

To analyze the effect of thermo-stability of free and immobilized Aly08, the enzymes were incubated at 37 °C and 45 °C for various times (0–90 min), respectively. As shown in Figure 4A, the free Aly08 rapidly lost 60% of its initial activity after incubation at 37 °C for 40 min. However, the immobilized AL-LMW-CS-NPs retained 76.8% of its initial activity even after incubation for 1 h at the same temperature. These results indicated that immobilized Aly08 showed more stability at 37 °C, which may induce a better anti-biofilm activity as the anti-biofilm activities determination always performed at 37 °C. The optimal temperatures of the free Aly08 and immobilized AL-LMW-CS-NP were 45 °C, so the thermal stability of the two enzymes were also analyzed at 45 °C. As shown in Figure 4B, immobilized AL-LMW-CS-NPs was also more stable than free Aly08. It may be hypothesized that the enhanced thermo-stability for the immobilized AL-LMW-CS-NPs may associate with some enzyme molecules entrapped into the core of nanoparticles, which could provide more rigid external backbone for enzyme molecules and protect the enzymatic configuration from distortion or damage by temperature exchange [33].

In previous studies, alginate lyases showed the disadvantage of poor thermal stability, which need to be rationally designed to improve their stability at different temperatures. Strategies to improve the thermal stability of alginate lyase have been reported, including physical, chemical, and biological methods. For example, the introduction of disulfide bonds by Yang et al. enhanced the stability of the alginate lyase cAlym derived from *Microbulbifer* sp. at 45 °C [34]. However, how to select a suitable disulfide bond which simultaneously increasing thermal stability without changing its secondary structure and not reducing its activity is a major problem. Moreover, several studies have shown that adding a certain amount of stabilizer in the enzyme solution is also conducive to the thermal stability of the enzyme, while Yang et al. promoted the stability of an alginate lyase AlyM after incubation at 40 °C for 2 h by adding 30% glycerol solution [35]. The presence of stabilizers has certain disadvantages, which complicates the purification and lyophilization of enzymes. In all transformation strategies, the use of CS-NPs to immobilize the enzyme has attracted more attention due to its unique properties. In this study, enzyme immobilized in LMW-CS-NPs can retained most of the original activity of free Aly08, and it can significantly improve the thermal stability of Aly08 at 37 °C and 45 °C for different times, which is beneficial to preservation of alginate lyase and its application in medicine of alginate lyases.

**Figure 5:**
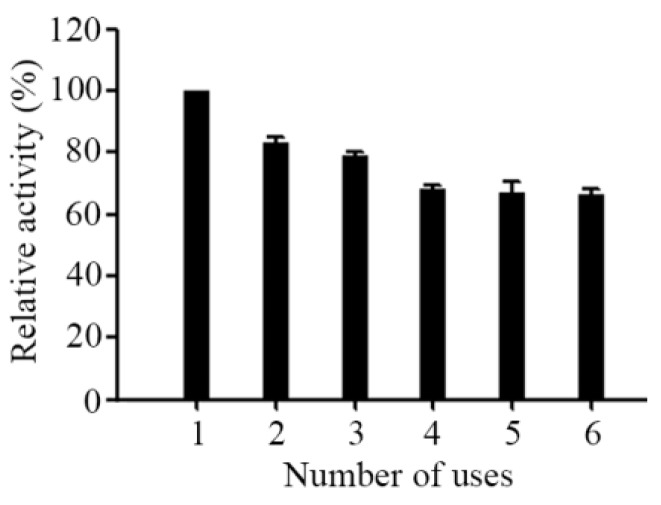
Reusability of immobilized Aly08. Activity for each cycle was compared with the initial activity.

Reusability can be defined as multi-use of the enzyme with the activity does not change much and it shown to be an important index for immobilized enzyme [36]. The main advantage of immobilized strategy is that it can increase the reusability of the enzyme preparation. In this study, we also investigated the reusability of immobilized AL-LMW-CS-NPs out of the awareness of environmental protection as well as saving costs as the reusability is essential for cost effective use in continuous industrial processes and in drug delivery devices. The immobilized AL-LMW-CS-NPs were washed with distilled water, and then added to a substrate solution to start every new cycle. As shown in Figure 5, there was a steady decline in the activity of the immobilized AL-LMW-CS-NPs within six cycles and it retained its relative activity over 60% of the initial activity at the end of the sixth cycle, which showed a good reusability for immobilized AL-LMW-CS-NPs. As a control, free Aly08 only use once. In summary, enzyme immobilization helped to resist chemical denaturation, improved stability and performed its reuse. The LMW-CS-NPs loaded Aly08 showed superb reusability, which shed light on its clinical application prospect in future.

### 2.3. Anti-Biofilm Activity of Immobilized AL-LMW-CS-NPs

**Figure 6:**
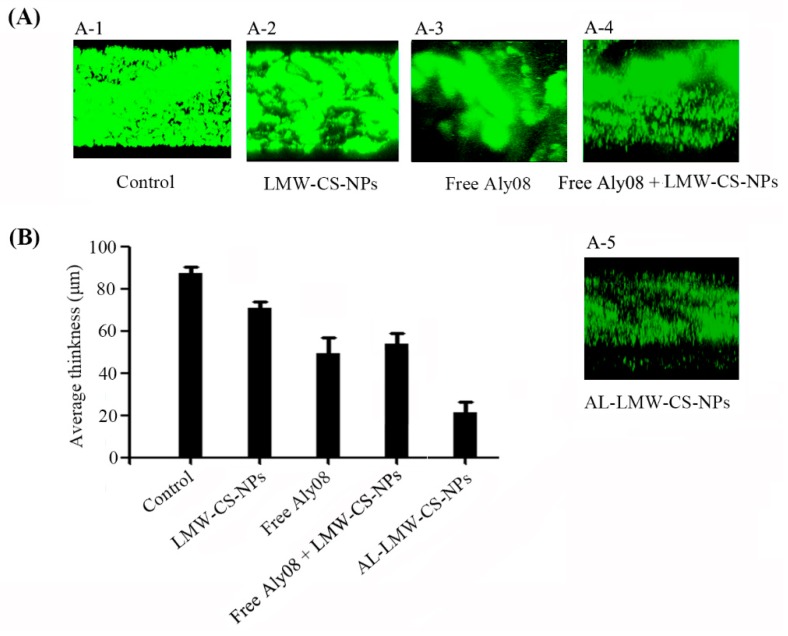
Prevention of biofilm formation in flow cell model. (**A**) Confocal laser scanning microscopy (CLSM) analysis of *P. aeruginosa* PAO1 biofilm formations in flow cell with media supplemented without (**A-1**) and with LMW-CS-NPs (**A-2**), free Aly08 (**A-3**), free Aly08 + LMW-CS-NPs (**A-4**) and AL-LMW-CS-NPs (**A-5**); (**B**) Average thickness of *P. aeruginosa* biofilms after indicated treatment.

To determine the preventing activity of immobilized AL-LMW-CS-NPs in *P. aeruginosa* biofilm formation, a flow cell model was established that allowed the continuous flow of fresh nutrients into a chamber. The *P. aeruginosa* PAO1 with pMRP 9-1 plasmid was grown in a “once-through” flow cell in the absence or continued presence of LMW-CS-NPs, free Aly08, or AL-LMW-CS-NPs. The biofilm formation was subsequently analyzed using confocal laser scanning microscopy (CLSM) (LSM 700, Carl-Zeiss, Germany). As shown in Figure 6A, the LMW-CS-NPs without Aly08 also showed slightly anti-biofilm activity. Compared with the free Aly08, the immobilized AL-LMW-CS-NPs significantly reduced the bacterial biomass in biofilm. Subsequently, the CLSM images were also analyzed by COMSTAT software. The average thickness of the formed biofilm without drug treatment was 87.6 ± 3.2 μm. The thickness of biofilm treated with AL-LMW-CS-NPs (21.7 ± 5.1 μm) was much weaker than treated with free Aly08 (49.6 ± 7.3 μm). These results indicated that the alginate lyase Aly08 immobilized on CS-NPs significantly increased biofilm inhibitory activity towards *P. aeruginosa.* Furthermore, the effect of the mixture of free Aly08 + LMW-CS-NPs was also determined (Figure 6A-4). Compared with the immobilized AL-LMW-CS-NPs, the group of free Aly08 + LMW-CS-NPs showed weaker anti-biofilm activity. The biofilm of free Aly08 + LMW-CS-NPs treatment (57.6 ± 5.0 μm) was even thicker than that treated with free Aly08 alone (49.6 ± 7.3 μm).

**Figure 7:**
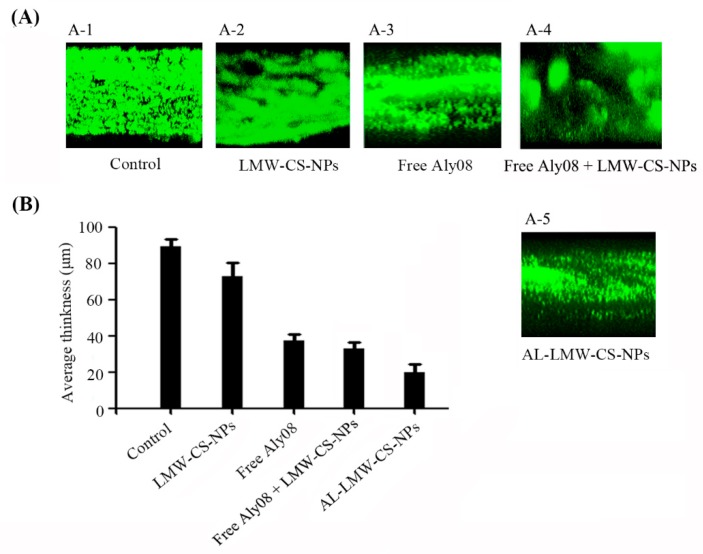
Biofilm disruptive activity in flow cell model. (**A**) CLSM analysis of *P. aeruginosa* PAO1 biofilm in flow cell with media supplemented without (**A-1**) and with LMW-CS-NPs (**A-2**), free Aly08 (**A-3**), free Aly08 + LMW-CS-NPs (**A-4**) and AL-LMW-CS-NPs (**A-5**); (**B**) Average thickness of *P. aeruginosa* PAO1 biofilm.

The immobilized AL-LMW-CS-NPs exhibited an inhibitory activity against bacterial biofilm construction. Simultaneously, whether the established biofilms were also subtle to AL-LMW-CS-NPs aroused our interest and attention. The experiment was carried out by incubating the strains in a flow-cell model and poured with fresh medium to form mature bacterial biofilms. Subsequently, the bacterial biofilms were transferred to medium supplemented with LMW-CS-NPs, free Aly08, or AL-LMW-CS-NPs, respectively. And then, the biofilms were analyzed by CLSM. As shown in Figure 7A, the pre-existing biofilms of *P. aeruginosa* were disrupted severely when treated with AL-LMW-CS-NPs. Meanwhile, LMW-CS-NPs without Aly08 could slightly disrupt the mature biofilm. Moreover, the effect of free Aly08 + LMW-CS-NPs was similar with the group of free Aly08 alone. Compared with the free Aly08, Aly08 immobilized on CS-NPs significantly reduced the bacterial biomass in biofilm. Subsequently, the thickness analysis also indicated that the alginate lyase Aly08 immobilized on CS-NPs significantly increased the biofilm disruption activity (Figure 7B).

Alginate, a component abundantly existed in *P. aeruginosa* biofilm matrix, has been widely regarded as an excellent target for bacterial infections control. Alginate lyase degrade alginate, then further degrade the biofilm matrix of *P. aeruginosa*. However, there are little researches associated to the anti-biofilm activity of alginate lyase, although numerous alginate lyases have been isolated, identified and characterized. Previous anti-biofilm study was focused on the alginate lyase, AlgL, from *P. aeruginosa*, which play an important role in the alginate synthesis [37]. Further research indicated that alginate lyase produced by other bacteria also showed the similar activity in destroying *P. aeruginosa* biofilms, mainly the enzymes classified in polysaccharide lyase family 5 (PL-5) [38,39]. Most of the enzyme for anti-biofilm analysis are AlgL from Sigma-Aldrich. Recently, AlgL immobilized chitosan nanoparticles (CS-NPs) of ciprofloxacin has showed improved antimicrobial activity against the biofilm associated mucoid *P. aeruginosa* infection in CF patients [21]. In this study, an alginate lyase, Aly08, belonging to PL family 7, previously reported in our lab showed anti-biofilm activity towards *P. aeruginosa* PAO1 (Figure 6 and Figure 7). Compared with AlgL, Aly08 has a higher specific activity, which has better anti-biofilm activity towards *P. aeruginosa*. However, the primary limitation of application of this enzyme is its instability. To further enhance its application prospect, free Aly08 was immobilized on LMW-CS-NPs. The immobilized enzyme showed better thermo-stability and reusability than free enzyme. Moreover, the mixture of free Aly08 + LMW-CS-NPs could not achieve the same anti-biofilm effect compared with the immobilized enzyme. These results indicated that the immobilization played important role in improving anti-biofilm activity of alginate lyase.

### 2.4. AL-LMW-CS-NPs Increases the Antibiotics Susceptibility of P. aeruginosa Biofilm

Pathogenic bacteria with bacterial biofilms induce severe antibiotic resistance, which is a serious threat to human health. To investigate whether the Aly08-mediated biofilm disruption increased the antibiotic sensitivity of *P. aeruginosa* biofilm, the effects of minimum inhibitory concentration (MIC) and minimum biofilm eradication concentration (MBEC) of antibiotics were examined using piperacillin, ceftazidime, and amikacin, respectively. As shown in Table 1, *P. aeruginosa* PAO1 biofilm at 96-well model needed high concentration of antibiotics to kill *P. aeruginosa* biofilm (MBEC) by piperacillin (>2048 mg/mL), ceftazidime (2048 mg/mL), and amikacin (1024 mg/mL). When treated with free Aly08, the *P. aeruginosa* PAO1 biofilm was obviously sensitive towards antibiotics, such as piperacillin (512 mg/mL), ceftazidime (256 mg/mL), and amikacin (256 mg/mL). This result indicated that Aly08-mediated biofilm dispersion increased the antibiotics’ capability of killing bacterial biofilms by indirectly targeting to bacterial cells. When the biofilm treated with LMW-CS-NPs, the nanoparticle itself only slightly enhanced its antibiotic sensitivity. Moreover, the synthetic AL-LMW-CS-NPs could obviously enhance the antibiotic sensitivity of *P. aeruginosa* PAO1, such as piperacillin (256 mg/mL), ceftazidime (64 mg/mL) and amikacin (64 mg/mL) (Table 1). These results indicated that the alginate lyase Aly08 immobilized on CS-NPs significantly increased antibiotic sensitivity towards *P. aeruginosa* biofilms.

The existence of biofilm network prevents the penetration of the antimicrobial agent which is one of the most important reasons for the development of antibiotic-resistant bacteria [40]. Matrix-targeting enzymes, such as alginate lyase, were applied against multi-resistant *P. aeruginosa* infection. The permeability and fluidity of antibiotics were relatively low in the biofilm matrix, which resulting in high tolerance of bacteria to antibiotic treatment. In a previous study, alginate lyase combined with antibiotics could be used in enhancing the disruption of *P. aeruginosa* biofilm through the degradation of alginate components in biofilm EPS, allowing antibiotics embedded the bacterial colonies with higher concentrations [38]. The combination of different antibiotics (piperacillin, ceftazidime, and amikacin) with AL-LMW-CS-NPs has been tried to disrupt *P. aeruginosa* biofilms in this study, and it significantly improved antibiotic activity and antibiofilm activity due to the direct destruction of biofilm. Further research is needed orienting to in vivo studies.

## 3. Materials and Methods

### 3.1. Materials

High molecular weight chitosan (Mw: 140–190 kDa, degree of deacetylation: > 85%, the viscosity: 200–400 mPa.s, cat. no. C105802) was purchased from Aladdin, Shanghai, China. *N*-(3-Dimethylaminopropyl)-*N*’-ethylcarbodiimide hydrochloride (EDAC), *N*-Hydroxysuccinimide (NHS) and Tri-polyphosphate (TPP) were purchased from Macklin, China; Piperacillin, Ceftazidime and Amikacin were purchased from Sigma-Aldrich, St. Louis, MO, USA. All other chemicals used in this study were of analytical grade.

### 3.2. Preparation of Low Molecular Weight Chitosan

The chitosanase, CsnM, used in this study was purified in our lab [32]. The purchased HMW-CS was dissolved in 1 M aqueous acetic acid (HAc) to a concentration of 1% (*w/v*), and the pH was adjusted to 6.0 using 1 M NaOH. To get the LMW-CS, 1 mL of CsnM (20 U/mL) was added to 200 mL of colloidal chitosan at 20 °C for 10 min in a shake condition. Then, the reaction was heated at 100 °C for 10 min and centrifuged for 10 min at 10,000 g to remove the enzyme and undegrade chitosan. Then, the degrading product was dialyzed by a 1000 Da dialysis bag to remove smaller chitosan oligosaccharides. The average molecular weight of LMW-CS was performed using viscosity methods [41].

### 3.3. Synthesis of Chitosan Nanoparticles

The technique of ionotropic gelation was used for preparing the chitosan nanoparticles (CS-NPs) [21]. Concisely, a clear 0.1% (*w/v*) chitosan solution was prepared by adding LMW-CS in 1 mol/L acetic acid (pH 6.5) followed by continues stirring for 2 h. Then 0.1% (*w/v*) polyanionic cross-linking agent, TPP, was slowly added to the prepared chitosan solution in a ratio of 1:4 while stirring at a room temperature under vigorous magnetic stirring in order to form low molecular weight chitosan nanoparticles (LMW-CS-NPs). Then, the formed LMW-CS-NPs were collected by centrifuged twice at 10,000 g for 20 min using 10 µL glycerol bed. To immobilize the free Aly08, on CS-NPs, 100 mg/mL of Aly08 was firstly activated with a mixed solution with EDAC (0.1 M) and NHS (0.1 M) followed by stirring at 10 °C for 30 min. The formation of the alginate lyase immobilized chitosan nanoparticles (AL-LMW-CS-NPs) could be achieved by adding 0.05–0.50 mg/mL of the final activated Aly08 concentrations to 1 ml LMW-CS-NPs solution (0.2 mg/mL), following by sonicating the suspension and ultrafiltrating with the Centricon centrifugal filter devices (molecular weight cut-off 50 kDa, Millipore). Moreover, the LC and LE% of AL-LMW-CS-NPs were determined as shown in following equations, respectively:

(1) $$\mathrm{LC}\;=\;\frac{\left(A-B\right)}{C}$$

(2) $$\mathrm{LE}\%\;=\;\frac{\left(A-B\right)}{A}\times 100\%$$

A and B represent the amount of initial and unload Aly08, respectively, whereas C represents the weight of the nanoparticles. All assays were performed in three parallel experiments and showed the data as mean ± standard deviation.

### 3.4. Analysis of the Particle Characterization 

The prepared AL-LMW-CS-NPs were characterized by Transmission electron micrograph (TEM) (JEM-2100, JEO, Japan). In order to investigate both the shape and surface morphology of the immobilized AL-LMW-CS-NPs, the samples were dried with a single drop on a copper foil or carbon substrate and following by gold coated. TEM observations were done to obtain the image at an accelerating voltage of 15.0 kV [17].

The quantification of amino group in the surface of CS-NPs was determined spectrophotometrically by a method of modified Ninhydrin assay in order to identify the conjugation [42]. Briefly, a small aliquot of beads was smeared on filter paper, sprayed with ninhydrin solution (0.2% (*w/v*) in acetone), and heated briefly with a hair dryer. The appearance of purple color indicated the presence of free amino groups, whereas the color disappearance indicated that enzymes had been linked to the amino groups. The cross linked HMW-CS-NPs, LMW-CS-NPs and AL-LMW-CS-NPs (50 µg/mL) with the mixtures were determined its absorbance at 570 nm (A570) using the Pearl-360 spectrophotometer (IMPLEN, Munich, Germany). Thus, the percentage of free amino group was using the following relation:

(3) $$\%\;\mathrm{Free}\;\mathrm{Amino}\;\mathrm{Group}\;=\;\frac{A570\;for\;chitosan\;in\;LMW-CS-NPs/Al-LMW-CS-NPs}{A570\;for\;chitosan\;in\;LMW-CS-NPs}\;\times \;100\%$$

Here, A570 represents the absorbance of Chitosan and Aly08 in each sample.

### 3.5. Enzyme Activity and Stability

The activity of free Aly08 and immobilized AL-LMW-CS-NPs was determined by A235 method reported in our previous study. Briefly, 100 µL enzymes were added to the 900 µL sodium alginate solution [0.3% (*w/v*) in 20 mM Glycine-NaOH buffer (pH 8.35)] for incubation at 45 °C for 10 min. The enzyme activity of one unit (U) was the amount of added enzyme required that increases the absorbance at 235 nm by 0.1 per minute [43].

The effects of temperature on AL-LMW-CS-NPs and free Aly08 were evaluated by determining the activities at different temperatures (15–65 °C). The measurement of thermal stability is determined its residual activities after leaving it at 37 °C or 45 °C for various times (0–90 min). The optimum pH was also calculated with the enzyme incubating at different buffers. All reactions were performed in triplicate.

### 3.6. Anti-Biofilm Activity

Flow cell method was used to observe the in vitro anti-biofilm activity of LMW-CS-NPs, free Aly08, AL-LMW-CS-NPs, free Aly08 + LMW-CS-NPs, against *P. aeruginosa* PAO1 with pMRP 9-1 plasmid [18]. Dynamic biofilm formation was performed in “once-through” flow cells (4.5 mm × 2 mm × 35 mm). Briefly, each flow cell was injected with 0.8 ml of the diluted culture of test strain, and incubated at room temperature for 40 min without flow. Flow was started at a constant rate of 0.5 mL/min. The images were obtained using a confocal laser scanning microscope (CLSM) (LSM 700, Carl-Zeiss, Germany). Images were recorded at the green channel (excitation 488 nm and emission 518 nm) [19]. The images were constructed with the LSM 5 image browser.

### 3.7. Antibiotics Susceptibility Assay of Biofilm

MIC and MBEC analysis were performed as previously described [18,44]. Briefly, two-fold broth dilution method was determined to find the MIC of Piperacillin, Ceftazidime and Amikacin, as well as LMW-CS-NPs, free Aly08, free Aly08 + LMW-CS-NPs, AL-LMW-CS-NPs with these three antibiotics against *P. aeruginosa* PAO1. The test antibiotic was diluted with LB medium from 2048 mg/ml to 2 mg/mL by two-fold serial dilutions. Then bacterial culture (100 µL, 1 × 10^5^ CFU/mL) was seeded in the 96 well culture plate and incubated at 37 °C for 24 h with 100 µL of different test samples including two-fold concentration free antibiotics as well as LMW-CH-NPs, free Aly08, free Aly08 + LMW-CS-NPs, AL-LMW-CH-NPs. Eventually, inhibition of bacterial growth was recorded by measuring the absorbance values of tested samples on the multi-mode plate reader (SYNERGY/HTX, Biotek) at 600 nm. MBEC for different samples was evaluated using the biofilm grown on the peg lid. Briefly, 96 well culture plate filled with 200 µL, 1 × 10^5^ CFU/mL of *P. aeruginosa* PAO1 bacterial culture in LB medium was covered with peg lid and incubated at 37 °C for 48 h on a rocking shaker. Biofilms were formed on the pegs surfaces protruding down from the lid which suited the standard 96-well culture plates. After that, the peg lid was covered over the 96 well plate containing various tested samples, and each sample was incubated at the same temperature for 24 h. The system was incubated for 24 h at 37 °C. After 24 h, peg lids were removed and placed on the second 96 well plate containing fresh LB medium for sonication to remove the adherent biofilm.

## 4. Conclusions

In this study, we have demonstrated the successful immobilization and stabilization of alginate lyase, Aly08, on LMW-CS-NPs, and the immobilization improved the thermal stability and reusability of alginate lyase dramatically. This study also showed that the immobilized enzyme was more effective than free enzyme in inhibiting biofilm formation and interrupting the mature biofilm towards *P. aeruginosa*. In addition, the biofilm disruption by AL-LMW-CS-NPs greatly increased the antibiotic sensitivity of *P. aeruginosa* biofilm, which can be applied as a kind of excellent carrier for anti-biofilm agents. Our further work will be focused on in vivo analysis of the AL-LMW-CS-NPs, which will greatly enrich its application prospects.

## Figures and Tables

**Table 1:** Antibiotic sensitivity of *P. aeruginosa* PAO1 as a planktonic population (minimum inhibitory concentration, MIC) and as a biofilm population (minimum biofilm eradication concentration, MBEC).

Concentration(mg/mL)	Piperacillin	Ceftazidime	Amikacin
MIC	32	16	16
MBEC	>2048	2048	1024
MBEC with LMW-CS-NPs	2048	1024	512
MBEC with free Aly08	512	256	256
MBEC with free Aly08 + LMW-CS-NPs	512	256	256
MBEC with AL-LMW-CS-NPs	256	64	64

## Supplementary Materials

Supplementary materials can be found at https://www.mdpi.com/1422-0067/20/18/4565/s1. Figure S1: Transmission electron micrograph (TEM) analysis of high molecular weight chitosan nanoparticles.

## Abbreviations

EPS	extracellular polymeric substance
CS-NPs	chitosan nanoparticles
CF	Cystic fibrosis
LMW-CS	low molecular weight chitosan
HMW-CS	high molecular weight chitosan
LMW-CS-NPs	low molecular weight chitosan nanoparticles
HMW-CS-NP	high molecular weight chitosan nanoparticles
AL-LMW-CS-NPs	alginate lyase immobilized low molecular weight chitosan nanoparticles
TEM	Transmission electron micrograph
CLSM	Confocal laser scanning microscopy
TEM	Transmission electron microscopy
LC	loading capacity
%LE	loading efficiency
MIC	minimum inhibitory concentration
MBEC	minimum biofilm eradication concentration
EDAC	*N*-(3-Dimethylaminopropyl)-*N*’- ethylcarbodiimide hydrochloride
NHS	*N*-Hydroxysuccinimide
TPP	Tri-polyphosphate
