# Supplementary material for: Enhancing the Thermo-Stability and Anti-Biofilm Activity of Alginate Lyase by Immobilization on Low Molecular Weight Chitosan Nanoparticles
Source: Int J Mol Sci. 2019 Sep 14;20(18):4565. doi: 10.3390/ijms20184565 (PMC6770906; doi:10.3390/ijms20184565)
Supplement: Supplementary file 1 [file ijms-20-04565-s001.pdf]

## Supplementary Materials

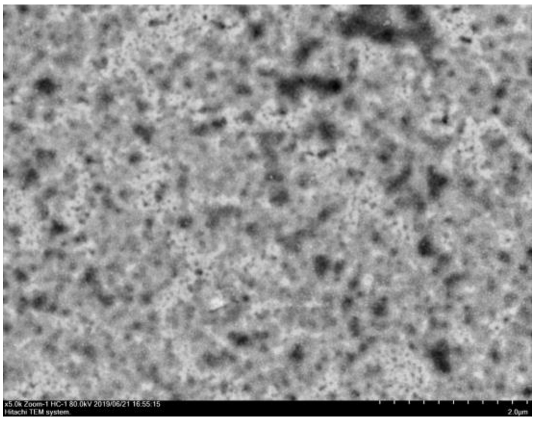


**Figure S1.** Transmission electron micrograph (TEM) analysis of high molecular weight chitosan nanoparticles.
